# A Global Overview of Per- and Polyfluoroalkyl Substance Regulatory Strategies and Their Environmental Impact

**DOI:** 10.3390/toxics13040251

**Published:** 2025-03-28

**Authors:** Ren-Shou Yu, Hui-Ching Yu, Ying-Fei Yang, Sher Singh

**Affiliations:** 1 Department of Tourism Management, Jimei University, Xiamen 361021, China; 201961000083@jmu.edu.cn; 2 Center for General Education, Cheng Shiu University, Kaohsiung 833301, Taiwan; k0299@gcloud.csu.edu.tw; 3 Department of Bioenvironmental Systems Engineering, National Taiwan University, Taipei 10617, Taiwan; 4 Department of Life Science, School of Life Science, College of Science, National Taiwan Normal University, Taipei 11677, Taiwan

**Keywords:** PFASs, persistence, bioaccumulation, ecotoxicity, jurisdiction, unknown or variable composition, complex reaction products or biological materials (UVCB)

## Abstract

Per- and polyfluoroalkyl substances (PFASs), while possessing desirable properties for human society, have increasingly raised concerns due to their environmental persistence, bioaccumulation, and ecotoxicity. One of the major challenges with PFASs is the inconsistent adoption of regulatory strategies by authorities across different countries and regions, making it difficult to address the issue on a global scale. To obtain a global overview of PFAS regulatory patterns, this study utilized the most recent PFAS regulatory databases across different jurisdictions, both local and global. Among all geographic regions, the United States Environmental Protection Agency (USEPA) and European Union (EU) Registration, Evaluation, Authorization, and Restriction of Chemicals (REACH) encompass the most jurisdictions for PFASs. However, most PFASs are without regulation under the current regulatory status. We also assessed the regulatory ecotoxicity status of PFASs under the Toxic Substances Control Act (TSCA) of the USEPA. The results showed that 36.3% of PFASs are of Unknown or Variable composition, Complex reaction products, or Biological materials (UVCB) and classified as E;P (persistent, bioaccumulative, and toxic), followed by 31.3% as P (persistent) and 13.2% as P;S (persistent and toxic). We highlight the regulatory patterns, industrial applications, and categorization of PFASs under different regulatory frameworks. The need for international cooperation and harmonized regulatory standards to mitigate PFAS pollution is also addressed. A coordinated effort involving regulatory agencies, industry, researchers, and the public will be essential to facilitate harmonized regulations of PFASs and ensure a sustainable and healthy environment.

## 1. Introduction

Per- and polyfluoroalkyl substances (PFASs) have emerged as a critical environmental and public health concern due to their persistence, bioaccumulation, and potential adverse effects [1,2,3]. These synthetic chemicals raise serious concerns because of their widespread use in various industrial and consumer products and their residuals in air, water, soil, and living organisms worldwide [4,5]. As scientific evidence increasingly links PFAS exposure to health issues such as cancer, thyroid disease, and immune dysfunction, regulatory frameworks are evolving globally to address this pervasive contamination [2,6]. Also, the chemicals can be transported over long distances, leading to the widespread contamination of habitats, and accumulate in the tissues of living organisms, magnifying in concentration as they move up the food chain [4,7]. Consequently, PFAS exposure in animals has been linked to reproductive and developmental issues, immune system impairment, and behavioral changes, impacting their survival and reproduction rates [6]. The ecological balance is also altered as PFASs affect the health and functioning of key species, leading to long-term consequences. These substantial threats necessitate urgent regulatory and remediation efforts to mitigate the impacts of PFASs on the environment [8].

Countries and regions, including the United States, the European Union, and Australia, are adopting relatively stricter regulations, implementing bans on specific PFAS compounds, setting stringent limits on PFAS levels in drinking water, and mandating extensive monitoring and reporting requirements [9,10,11]. This trend toward tighter regulation reflects a growing consensus on the need for comprehensive strategies to mitigate PFAS risks and protect public health and the environment. However, the regulatory landscape is complex and evolving, with countries adopting varying approaches to limit PFAS use, enforce cleanup efforts, and establish safety thresholds. This inconsistency complicates international trade and environmental protection efforts, as products containing PFASs might be regulated differently in neighboring jurisdictions. Harmonizing these regulations remains a global challenge, as does addressing the long-term ecological impacts of these persistent pollutants [12].

Additionally, the sheer number of PFAS chemicals—over 4700 known compounds with Chemical Abstracts Service (CAS) numbers on the global market and in the environment—poses a regulatory challenge [13,14,15,16,17]. Many countries struggle to keep pace with identifying and assessing the risks of each individual compound. This has led to a piecemeal regulatory approach, where some PFASs are regulated while others remain unaddressed, potentially allowing harmful substances to slip through regulatory gaps. Moreover, industry resistance and the economic implications of phasing out PFAS compounds add another layer of complexity. Industries reliant on PFASs for their unique properties may lobby against stringent regulations, citing the high costs and technical difficulties of finding suitable alternatives. This tension between economic interests and public health protection creates significant hurdles for policymakers [18].

Addressing PFAS contamination is constrained by technological and economic factors, including the high costs and technical challenges of remediation and the economic impact on industries reliant on PFASs. Current remediation technologies, such as activated carbon filtration and advanced oxidation processes, can be expensive and energy-intensive, limiting their widespread application [19]. Additionally, transitioning to PFAS-free alternatives requires significant investment in research and development, posing financial challenges for businesses. Researchers and industries are also exploring a range of substitutes that offer similar performance characteristics without the persistence and toxicity of PFASs [20]. These alternatives include fluorine-free firefighting foams, non-fluorinated coatings for textiles and cookware, and biodegradable materials for food packaging [21,22]. The successful adoption of PFAS alternatives depends on rigorous testing to ensure safety and efficacy, regulatory support to facilitate the transition, and industry commitment to sustainability and innovation. Therefore, in parallel with investments in remediation and finding alternatives for PFASs, identifying the regulatory status (referring to the classification and management of these substances based on international guidelines and regional regulations) in different regions and providing strategies for better engagement among authorities, industry, academia, or the public would help to mitigate the regional or global risks resulting from restrictions on/the banning of some PFASs in the future.

In summary, the regulatory challenge of PFASs worldwide stems from inconsistent standards, different definitions of PFASs, scientific challenges, and economic and industrial pressures. Addressing these issues requires a coordinated global effort, robust scientific research, and the development of safer alternatives to PFAS compounds. Therefore, the objectives of this study are fourfold: (i) to investigate the regulatory status of PFAS compounds in different regulatory databases, (ii) to explore the environmental impacts of PFASs under different authoritative criteria, (iii) to understand the industrial use pattern and the potential impacts of regulation, and (iv) to provide suggestions on long-term strategies for harmonizing regulatory conditions worldwide to mitigate environmental risks.

## 2. Materials and Methods

### 2.1. Data Sources and Investigated Jurisdictions

The database was originally the result of efforts by the OECD/UNEP Global PFC Group between January 2017 and February 2018 in updating the OECD’s “Lists of PFOS, PFAS, PFOA, PFCA, Related compounds and Chemicals That May degrade to PFCA” to provide a comprehensive list of PFASs that have been on the global market. The database was collated by utilizing publicly accessible information sources. The PFASs identified and included in the data collection by the OECD [23] have been specified in the document. In summary, there are commonly recognized groups of PFASs with established general terminology determined by Buck et al. [24]. New groups of PFASs fulfilling the common definition of PFASs (i.e., containing at least one perfluoroalkyl moiety) have also been identified in the data collection [24], including the following: (1) hydrofluorocarbons (HFCs) with a general structure of CnF2n+1CmH2m+1, hydrofluoroethers (HFEs) with a general structure of CnF2n+1OCmH2m+1, and hydrofluoroolefins (HFOs) with a general structure of CnF2n+1CmH2m-1, (2) perfluoroalkyl alkenes (CnF2n) and their derivatives, (3) perfluoroalkyl ketones (CnF2n+1C(O)CmF2m+1), semi-fluorinated ketones (CnF2n+1C(O)CmH2m+1), and their derivatives, (4) side-chain fluorinated aromatics (CnF2n+1−aromatic ring(s)), and (5) others such as perfluoroalkyl alcohols (CnF2n+1OH), silanes (CnF2n+1Si−), and amines (CnF2n+1−N−).

In total, 4730 PFAS-related CAS numbers have been identified and manually categorized, including new groups of PFASs that fulfill the common definition of PFASs (i.e., containing at least one perfluoroalkyl moiety). The selection of PFASs from individual sources was based on a decision tree, wherein only substances with a CAS No. containing a perfluoroalkyl moiety with three or more carbons (i.e., –CnF2n–, n ≥ 3) or a perfluoroalkylether moiety with two or more carbons (i.e., –CnF2nOCmF2m−, n and m ≥ 1) were selected. The compilation of PFASs was conducted by using the CAS No. as the unique substance identifier. The criteria for the categorization of individual PFASs include the following: (1) regulatory registration status; (2) structure category; (3) number of functional groups—mono- or multi-functional; (4) chain length of the “−CnF2n−” or “−CnF2nOCm F2m−” moiety; (5) linear or branched/cyclic isomer(s); (6) (potential) degradability in the environment and biota; (7) non-polymer(s) vs. polymers; (8) single substance vs. a mixture of substances; (9) related substances (e.g., monomers, acid form of a salt); and (10) uncertainty of the categorization [23].

We collected data on global regulatory patterns (referring to the various approaches and measures that different countries and regions have implemented to manage and control these chemicals) of PFASs from various sources of regulatory bodies, including databases from general national inventories (3_overview_with_CAS), inventories for specific uses (4_4_us_epa_cdr_2016), regulatory-specific lists for PFASs (4_0_us_epa_tsca, 4_4_us_epa_cdr_2016, 5_ca_dsl), and other scientific databases (3_overview_with_CAS) collated by the Organisation for Economic Co-operation and Development (OECD) [25]. Data collection also involved consulting industry reports and the scientific literature to gather comprehensive information on PFAS regulations across different regions [25]. To analyze the global regulatory trends, we used a combination of qualitative and quantitative methods by mapping out the regulatory patterns in different jurisdictions, distinguishing between different regulatory approaches and geographic regions.

Specifically, the jurisdictions investigated at a global scale include the following: (i) the OECD 2007 List (Lists of PFOS, PFAS, PFOA, PFCA, Related Compounds and Chemicals That May Degrade to PFCA (https://one.oecd.org/document/ENV/JM/MONO(2006)15/en/pdf, accessed on 18 November 2024), which includes substances from responses to OECD surveys, listed on the inventories of OECD member countries (with a perfluorinated chain of four or more carbons), reviewed by international agencies or regulatory bodies, and identified from published research; and does not contain chemical substances listed in the confidential sections of the inventories, the provisional PMN list of the TSCA and inventories of non-OECD countries, chemical intermediates and R&D chemicals, chlorofluorocarbons (CFCs), hydrofluorocarbons (HFCs), hydrochlorofluorocarbons (HCFCs), perfluorocarbons (PFCs), and polymers with fluorinated backbone structures (e.g., fluoropolymers), (ii) SciFinder (a scientific search engine and database that provides integrated access to the world’s most comprehensive and authoritative source of references, substances, and reactions in chemistry and related sciences, maintained by the Chemical Abstracts Service, a division of the American Chemical Society (https://scifinder.cas.org); the OECD used C2F5−, C3F7−, CF3O−, C2F5O−, C3F7O−, iso-C3F7−, and iso-C3F7O− as sub-structures for the substance search, and selected substances that have any references (e.g., peer-reviewed articles, patents, etc.) and commercial sources listed in SciFinder for further PFAS identification; accessed by OECD [25] on 9 February 2017)), and (iii) KEMI (Occurrence and use of highly fluorinated substances and alternatives (https://www.kemi.se/global/rapporter/2015/report-7-15-occurrence-and-use-of-highly-fluorinated-substances-and-alternatives.pdf, accessed on 22 March 2025), based on information from databases that the Swedish Chemicals Agency has access to, information in scientific articles, various reports on, and lists of, industrial chemicals from other countries (mainly in North America and Asia), and information from industry; includes both PFASs and other highly fluorinated substances).

In addition, sources of PFAS jurisdictions in North America include the following: (i) US EPA TSCA 12b, which is the non-confidential portion of the US EPA’s Toxic Substances Control Act Chemical Substance Inventory for Chemicals subject to TSCA Section 12(b) Export Notification Requirements (https://www.epa.gov/tsca-import-export-requirements/chemicals-subject-tsca-section-12b-export-notification-requirements, accessed on 22 March 2025); (ii) the US FDA Inventory of Effective Food Contact Substances (FCS) (US FDA Inventory of Effective Food Contact Substance Notification (https://www.fda.gov/food/packaging-food-contact-substances-fcs/inventory-effective-food-contact-substance-fcs-notifications, accessed on 18 November 2024); effective premarket notifications for food contact substances that have been demonstrated to be safe for their intended use in the United States; the database is regularly updated and was last accessed by OECD [25] on 3 October 2017); (iii) the US EPA CDR (formerly IUR) database (US EPA Chemical Data Reporting (formerly Inventory Update Rule) Inventories (https://www.epa.gov/chemical-data-reporting, accessed on 22 March 2025); basic exposure-related information on the types, quantities, and uses of chemical substances produced domestically and imported into the United States when production volumes for the chemical are 25,000 pounds or greater at any single site for a specific reporting year (a reduced reporting threshold—2500 pounds—now applies to chemical substances subject to certain TSCA actions); data collected in 1986, 1990, 1994, 1998, 2002, 2006, 2012, and 2016) for the years 2012 and 2016; (iv) US EPA IUR 2006 (https://www.epa.gov/chemical-data-reporting/downloadable-2006-iur-public-database, accessed on 22 March 2025) and IUR (now CDR) 1986-2002 (previously downloaded from the US EPA website; the link is no longer provided), including primarily unregulated PFASs or those with minimal regulation; (v) the US EPA Toxic Substances Control Act (TSCA) Inventory (a list of each chemical substance that is manufactured or processed, including imports, in the United States for uses under the TSCA (https://www.epa.gov/tsca-inventory/how-access-tsca-inventory, accessed on 22 March 2025); regularly updated, and last updated in June 2017 (used in this study)); (vi) the Canadian Domestic Substances List (DSL) (Canadian Domestic Substances List (https://pollution-waste.canada.ca/substances-search/Substance?lang=en, accessed on 22 March 2025); includes substances manufactured in, imported into, or used in Canada on a commercial scale; regularly updated, and last accessed on 13 October 2017 for this study); and (vii) Canada PCTSR 2012 with a non-exhaustive list of Chemical Abstracts Service registry numbers for substances subject to the Prohibition Of Certain Toxic Substances Regulations, 2012; the list was kindly provided by the Government of Canada in November 2016. For Nordic countries, there is Substances in Preparations in Nordic Countries (SPIN) (http://spin2000.net, accessed on 22 March 2025; includes quantities, the industries in which the substances are used, and the function of chemicals that are used in Nordic countries based on data from the Product Registries of Norway, Sweden, Denmark, and Finland; regularly updated (last accessed on 29 November 2017 by the OECD [25])). In the European Union (EU), a majority of PFASs are unregulated, with a few regulated entries under EU REACH that are Pre-registered (used “fluoro” as the keyword based on the version corresponding to the last update on 10 May 2016; https://echa.europa.eu/information-on-chemicals/pre-registered-substances (accessed on 22 March 2025); the database is regularly updated and was last accessed by OECD [25] on 29 November 2017) and Registered (used “fluoro” as the keyword based on the version corresponding to the last update on 20 January 2017; https://echa.europa.eu/information-on-chemicals/registered-substances (accessed on 22 March 2025); the database is regularly updated and was last accessed by OECD [25] on 3 October 2017).

For countries in the Asia–Pacific, Japan has the Existing and New Chemical Substances Inventory (ENCS) (List of Existing and New Chemical Substances (http://www.nite.go.jp/en/chem/chrip/chrip_search/systemTop, accessed on 22 March 2025); regularly updated, and last accessed on 3 October 2017) and the Examples of PFOA Stockholm Convention with predominantly unregulated PFASs (based on the discussion of POPRC12 under the Stockholm Convention; prepared for a domestic survey by the Japanese METI; http://www.meti.go.jp/policy/chemical_management/int/2_besshi2.pdf, accessed on 22 March 2025). The China IECSC contains an inventory of existing chemical substances produced or imported in China (https://chemicalwatch.com/asiahub/15187/existing-chemical-substances-inventory-2016-revisionchina/, accessed on 22 March 2025; the database is periodically updated and was last accessed by the OECD [25] on 29 November 2017). Australia has the Australian Inventory of Chemical Substances (AICS) (https://services.industrialchemicals.gov.au/search-inventory/, accessed on 22 March 2025; a database of chemicals available for industrial use in Australia; regularly updated; a list of substances containing the term “fluoro” in the chemical name was kindly provided by the National Industrial Chemicals Notification and Assessment Scheme (NICNAS) on 3 October 2017) and Australian Inventory Multi-tiered Assessment and Prioritisation (IMAP) Tier 2 (substances that have been assessed at Tier 2 (individual chemical evaluation) under Inventory Multi-tiered Assessment and Prioritisation (IMAP) by the NICNAS for the human health and environmental impacts of unassessed industrial chemicals listed on the Australian Inventory of Chemical Substances (AICS) (https://files.chemicalwatch.com/imapreview.pdf, accessed on 22 March 2025); the list was kindly provided by the Government of Australia in December 2016).

Our analysis included evaluating listed PFASs flagged with different criteria, such as industry use, function, and type. This classification helped in understanding the industrial impact and compliance requirements. Additionally, we reviewed the literature to assess the environmental implications of PFASs, identifying key trends and gaps in current regulations. This study provides visual representations of our data analysis, including the mind mapping of regulatory patterns (https://xmind.ai/), jurisdictional comparisons, and detailed categorizations of PFASs based on their chemical properties and regulatory status. The tools used for data analysis and visualization include Microsoft Excel (Microsoft Corporation, Redmond, WA, USA, 2018; retrieved from https://office.microsoft.com/excel, accessed on 22 March 2025) and Python Software (Python Language Reference, version 3.12.4; available at http://www.python.org).

### 2.2. Study Framework

**Figure 1:**
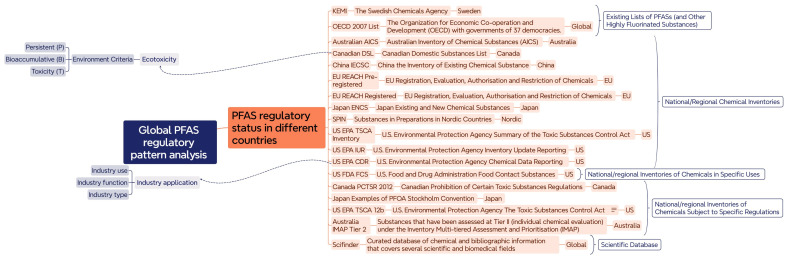
Illustration of a mind map to address the issue of global PFAS regulatory status encompassing the topics of regulatory status, ecotoxicity categorization, and industry application.

To illustrate the workflow of this study, we developed a study framework to streamline the rationale for addressing the global regulatory pattern of PFASs with a mind map (Figure 1). This mind map serves as a visual tool to understand the complex and multifaceted nature of PFAS regulation on a global scale, highlighting the interconnectedness of various regulatory elements and their impacts. This comprehensive diagram encompasses various associated topics, including regulatory practices in different regions, ecotoxicity, and categories in industry applications. The central theme of the mind map is the “PFAS regulatory pattern globally”, which branches into several key areas: (i) PFAS regulatory status on a national or global scale—this section maps out the regulatory status in various countries and regions, including Sweden (KEMI), Australia (AICS), Canada (DSL, PCTSR 2012), China (IECSC), Japan (ENCS, PFOA Stockholm Convention), the European Union (EU REACH Pre-registered, Registered), Nordic countries (SPIN), and the United States (TSCA Inventory, IUR, CDR, FDA FCS, TSCA 12b), and global databases (SciFinder, OECD 2007 List); (ii) ecotoxicity—this section highlights the environmental criteria such as persistence (P), bioaccumulation (B), and toxicity (T) that are considered in regulatory frameworks; and (iii) chemical production—this section addresses the industrial aspect, examining PFASs based on industry use, industry function, and industry type. It reflects the various ways PFASs are utilized in different sectors, which influences regulatory decisions.

## 3. Results

### 3.1. Global PFAS Regulatory Pattern

**Figure 2:**
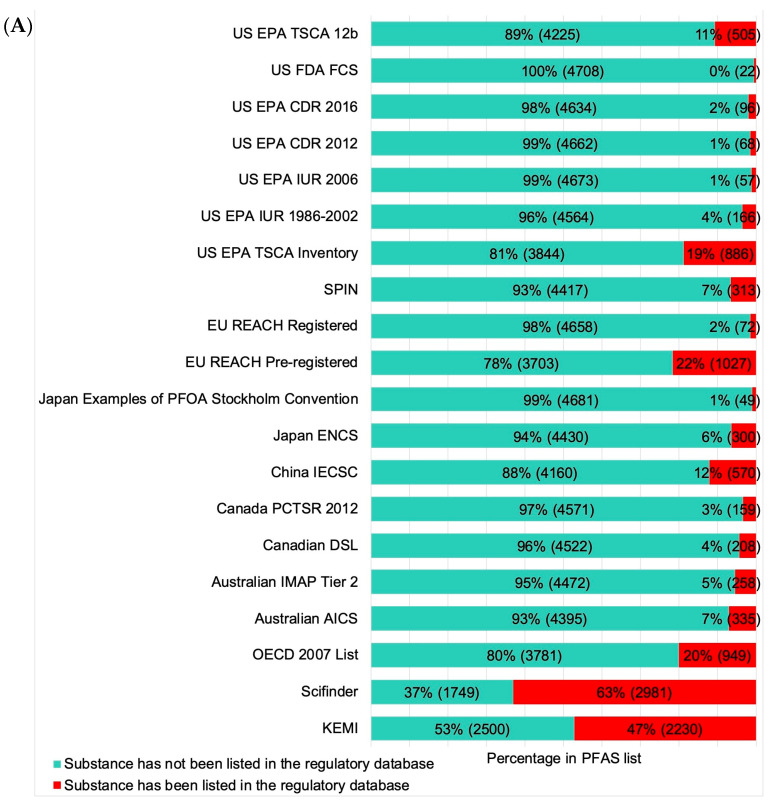

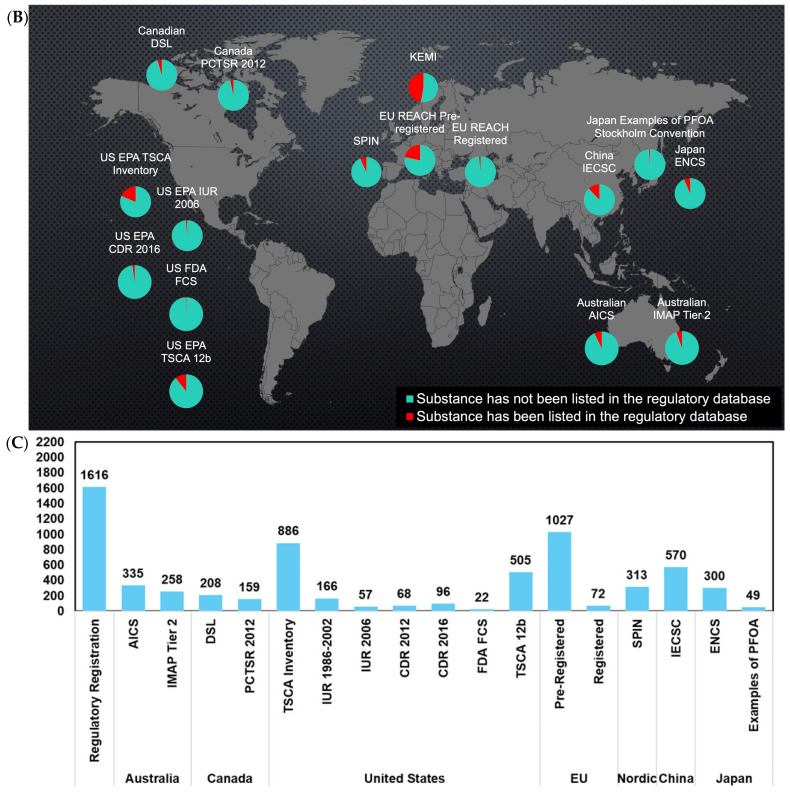
PFASs listed in different (**A**) regulatory databases, (**B**) databases corresponding to different geographic regions, and (**C**) PFASs with regulatory registration information in the SciFinder database.

Figure 2 illustrates the regulatory patterns of PFASs across different jurisdictions and geographic regions and the status of regulatory registration in the SciFinder database. This comprehensive analysis presents the percentage of PFAS entries that are regulated versus those that are not regulated in various international and national databases. The bar chart shows data from multiple regulatory sources, categorized by whether the PFASs listed in these sources are regulated (indicated in red) or unregulated (indicated in turquoise) (Figure 2A). This analysis helps in understanding the distribution and regulatory status of PFASs globally, highlighting the varying degrees of regulation across different regions and providing insights into how different jurisdictions handle PFAS management (Figure 2B). In addition, in the SciFinder database, around one-third of identified PFASs (1616 CAS numbers) have been listed under at least one national/regional chemical inventory, of which the EU REACH Pre-registered database has the highest number (1027) of PFASs with regulatory registration information in the SciFinder database, followed by the US EPA TSCA Inventory (886), the China IECSC (570), and the US EPA TSCA 12b (505) (Figure 2C).

### 3.2. PFAS Regulatory Categorization Under TSCA by USEPA

**Figure 3:**
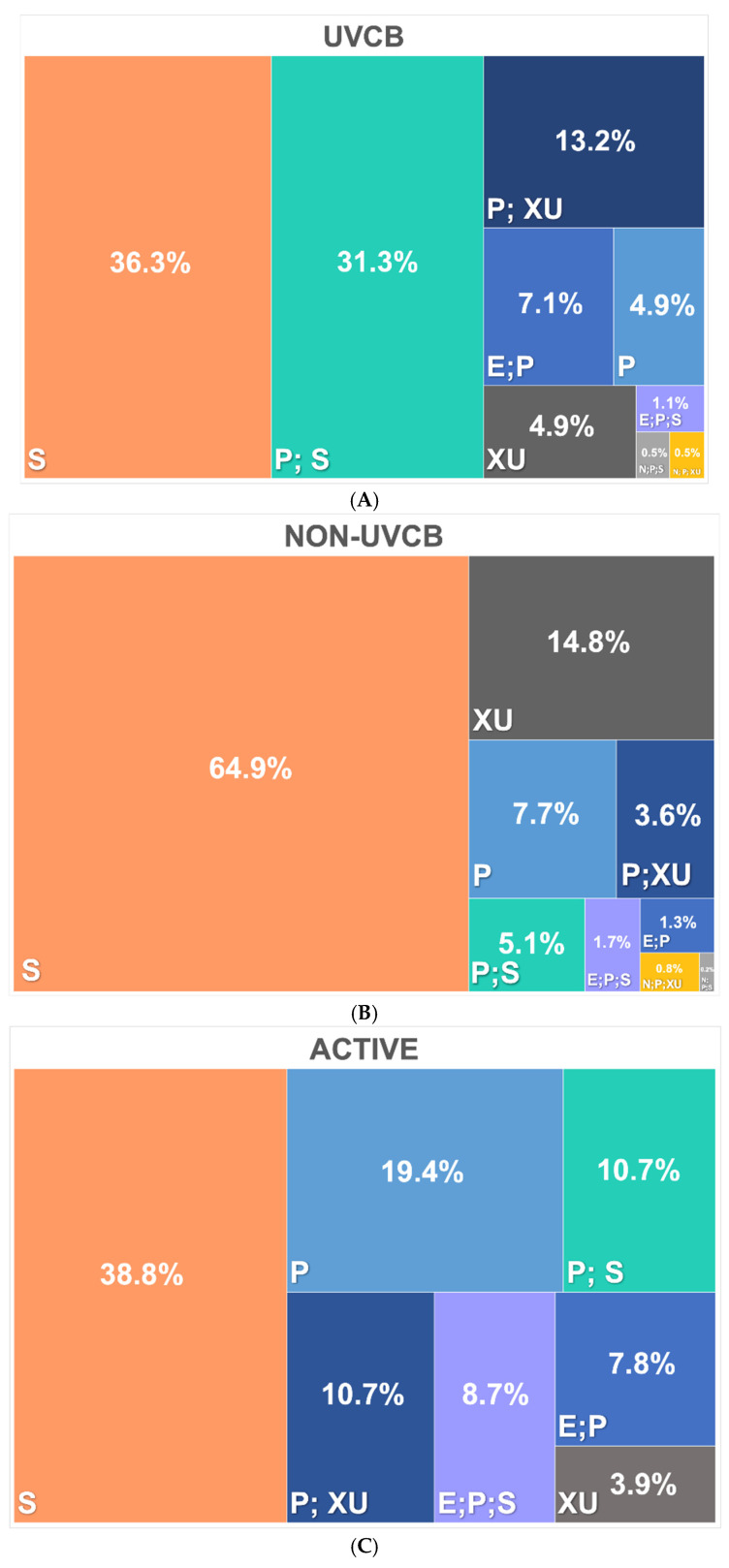

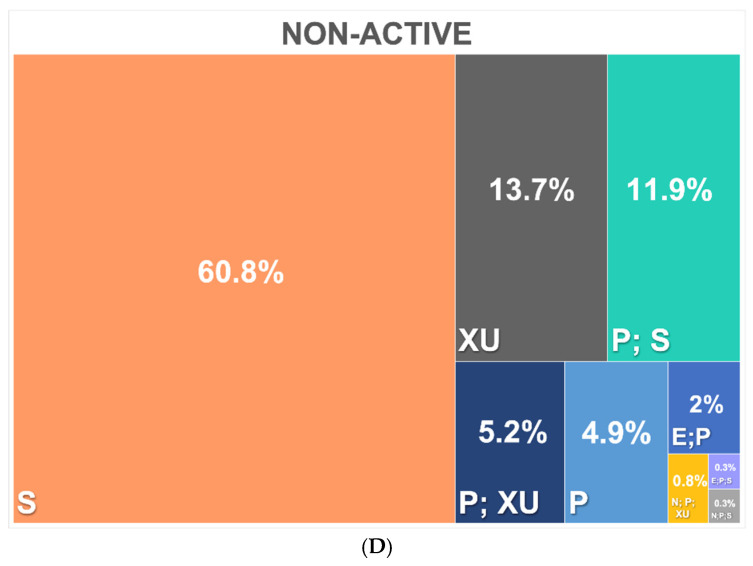
Listed PFASs flagged (**A**) as UVCB or (**B**) non-UVCB and (**C**) active or (**D**) non-active under the TSCA by the USEPA.

Figure 3 shows the PFAS regulatory categorization (referring to the categorization of PFASs by flagging them as UVCB or listing them as “active”) under the TSCA by the USEPA. The PFASs are classified based on their status as UVCB (Unknown or Variable composition, Complex reaction products, or Biological materials), non-UVCB, active, and non-active. The definition of active status is substances on the TSCA Inventory that can be manufactured, imported, or processed without any notifications. On the other hand, substances designated as “inactive” on the TSCA Inventory must be notified to the US EPA via a Notice of Activity Form (NOA-B) 90 days prior to manufacture, import, or processing. On receiving the notification form NOA-B, the EPA will change the designation of the notified chemical substance on the TSCA Inventory from inactive to active. Thus, the category in the TSCA database is of industry importance in the US region. Figure 3A illustrates PFASs under the UVCB category. The results showed that 36.3% of UVCB PFASs are classified as S (indicating a substance that is identified in a final Significant New Use Rule), 31.3% are classified as P;S (indicating a commenced Premanufacture Notice (PMN) substance (P) that is identified in a final Significant New Use Rule (S)), and 13.2% are classified as P;XU (indicating a commenced Premanufacture Notice (PMN) substance (P) that is exempt from reporting under the Chemical Date Reporting Rule (formerly the Inventory Update Reporting Rule), i.e., Partial Updating of the TSCA Inventory Data Base Production and Site Reports (40 CFR 711) (XU)). Smaller percentages fall into other categories such as E;P (7.1%; indicating a substance that is the subject of a Section 5(e) Consent Order under the TSCA (E) and a commenced Premanufacture Notice (PMN) (P)), P (4.9%; a commenced Premanufacture Notice (PMN) substance), XU (4.9%; indicating a substance exempt from reporting under the Chemical Date Reporting Rule (formerly the Inventory Update Reporting Rule), i.e., Partial Updating of the TSCA Inventory Data Base Production and Site Reports (40 CFR 711)), E;P;S (1.1%; indicating a substance that is the subject of a Section 5(e) Consent Order under the TSCA (E) and a commenced Premanufacture Notice (PMN) (P), and identified in a final Significant New Use Rule (S)), N;P;S (0.5%; indicating a polymeric substance that contains no free radical initiator in its inventory name but that is considered to cover the designated polymer made with any free radical initiator regardless of the amount used (N), is a commenced Premanufacture Notice (PMN) substance (P), and is identified in a final Significant New Use Rule (S)), and N;P;XU (0.1%; indicating a polymeric substance that contains no free radical initiator in its inventory name but that is considered to cover the designated polymer made with any free radical initiator regardless of the amount used (N), is a commenced Premanufacture Notice (PMN) substance (P), and is exempt from reporting under the Chemical Date Reporting Rule (formerly the Inventory Update Reporting Rule), i.e., Partial Updating of the TSCA Inventory Data Base Production and Site Reports (40 CFR 711) (XU)). For PFASs under category of non-UVCB, 64.9% are classified as S, followed by XU (14.8%), P (7.7%), P;S (5.1%), P;XU (3.6%), E;P;S (1.7%), E;P (1.3%), N;P;XU (0.8%), and N;P;S (0.2%) (Figure 3B).

For active PFASs, 38.8% are classified as S, 19.4% as P, and 10.7% as P;S and P;XU. Smaller percentages are classified into other combinations such as E;P;S (8.7%), E;P (7.8%), and XU (3.9%) (Figure 3C). For non-active PFASs, 60.8% are classified as S, 13.7% as XU, and 11.9% as P;S. Smaller percentages are classified into other combinations of non-active properties including P;XU (5.2%), P (4.9%), E;P (2%), N;P;XU (0.8%), E;P;S (0.3%), and (0.3%) N;P;S. These subfigures collectively show the distribution and regulatory status of PFASs under the TSCA framework, highlighting the prevalence of certain PFAS categories within the regulatory environment (Figure 3D).

### 3.3. PFAS Regulatory Categorization Under CEPA

**Figure 4:**
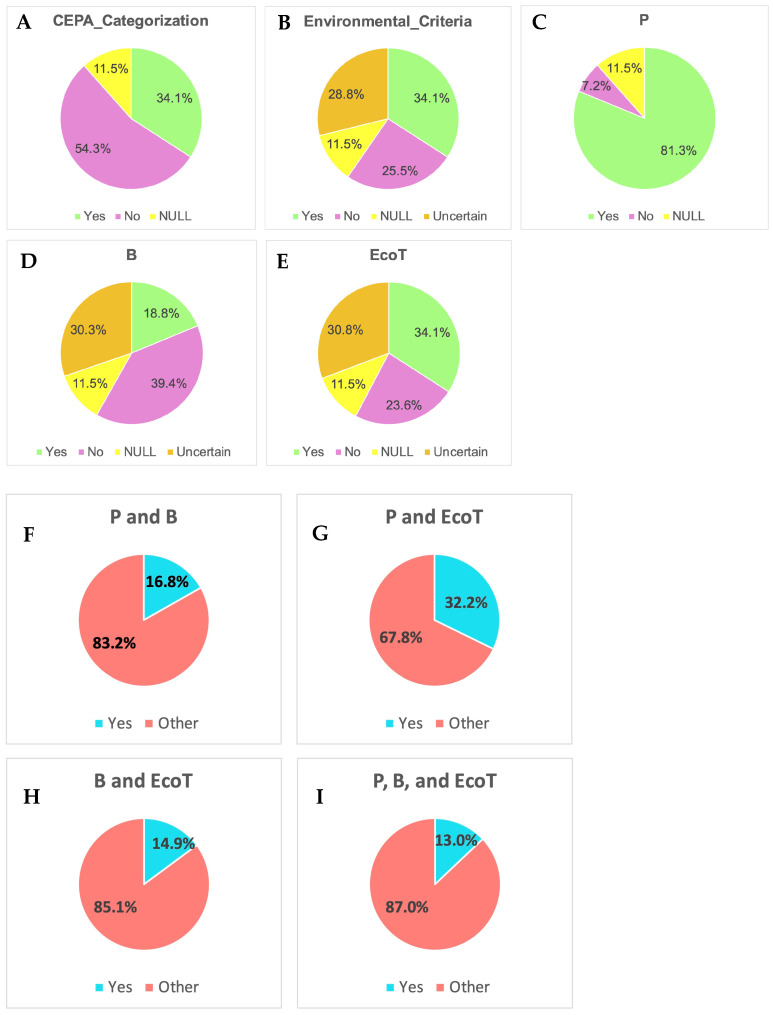
PFASs listed under different categories of (**A**) CEPA categorization and (**B**) environmental criteria and under (**C**) persistent (P), (**D**) bioaccumulative (B), (**E**) ecotoxic (EcoT), (**F**) P and B, (**G**) P and EcoT, (**H**) B and EcoT, and (**I**) P, B, and EcoT in the Canadian Domestic Substances List.

Figure 4 shows the categorization of PFASs under the CEPA in the Canadian Domestic Substances List, highlighting the environmental and toxicological criteria used to assess and manage these substances within Canada. PFASs are classified based on various criteria, including the environmental criteria of persistence (P), bioaccumulation (B), and ecotoxicity (EcoT). The data are presented in percentages for each category, showing the distribution of PFASs in relation to their categorization under the CEPA and the specific criteria. Under CEPA categorization, 34.1% of PFASs are categorized, 54.3% are not categorized, and 11.5% have a NULL categorization status (Figure 4A). For environmental criteria, 34.1% of PFASs meet environmental criteria, 25.5% do not meet these criteria, 28.8% have uncertain status, and 11.5% have a NULL status (Figure 4B). To better understand the regulatory patterns under different environmental criteria, we investigated the patterns under the categories of P, B, and EcoT. Under the category of P, 81.3% of PFASs are classified as persistent, 7.2% are not classified, and 11.5% have a NULL status (Figure 4C). For category B, 18.8% of PFASs are classified as bioaccumulative, 39.4% are not classified, 30.3% have uncertain status, and 11.5% have a NULL status (Figure 4D). For EcoT, the results showed that 34.1% of PFASs are classified as ecotoxic, 23.6% are classified as non-ecotoxic, 30.8% have uncertain status, and 11.5% have a NULL status (Figure 4E). Moreover, for PFASs under P and B, 16.8% are categorized as persistent and bioaccumulative and 83.2% fall under other categories. For P and EcoT, 32.2% are categorized as persistent and ecotoxic and 67.8% fall under other categories. For B and EcoT, 14.9% are categorized as bioaccumulative and ecotoxic and 85.1% fall under other categories. For P, B, and EcoT, 13.0% are categorized as persistent, bioaccumulative, and ecotoxic and 87.0% fall under other categories (Figure 4F–I).

### 3.4. PFAS Applications Based on Industry Parameters

**Figure 5:**
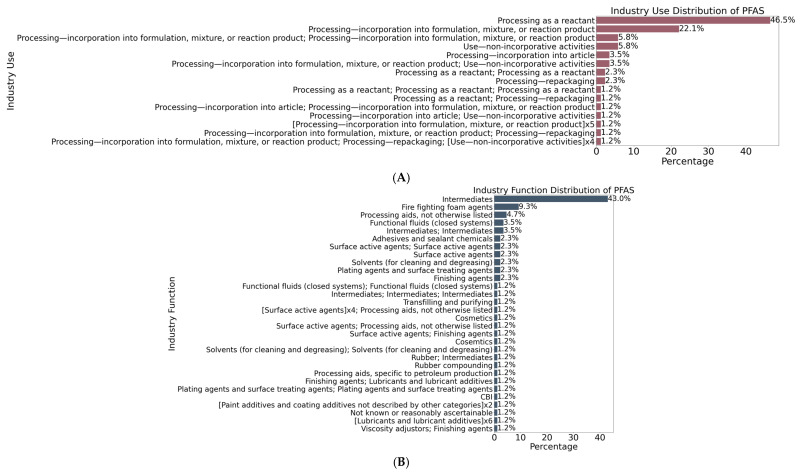

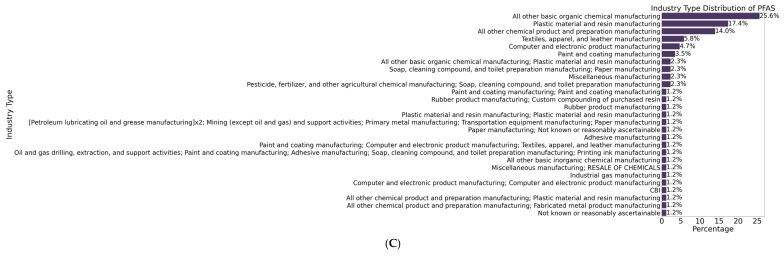
List of PFASs flagged with different (**A**) industry uses, (**B**) industry functions, and (**C**) industry types.

Figure 5 categorizes PFASs based on different industry-related parameters—industry use, industry function, and industry type—illustrating the prevalence and regulatory status of PFASs across various industrial applications. For PFAS application in various industrial processes, the majority of PFASs are used in processing as a reactant, accounting for 46.5% of the total usage, followed by PFASs used in processing—incorporation into formulation, mixture, or reaction product, which represents 22.1%. Other notable uses include withheld information (26.2%) and use—non-incorporative activities (5.8%) (Figure 5A). For the industry functions of PFASs, the largest category is intermediates, accounting for 43.0% of the total. Other significant functions include firefighting foam agents (9.3%), processing aids not otherwise listed (4.7%), and functional fluids in closed systems (3.5%). The distribution reveals that PFASs are predominantly used as intermediates, with other functions spanning a range of industrial applications including lubricants, sealants, and solvents (Figure 5B). For the distribution of PFASs across different industry types, the most significant category is “all other basic organic chemical manufacturing”, which comprises 25.6% of the total, followed by “plastic material and resin manufacturing” (17.4%) and “all other chemical product and preparation manufacturing” (14.0%). Other industry types include miscellaneous manufacturing, paper manufacturing, and textiles, apparel, and leather manufacturing. The figure highlights the diverse industrial sectors that utilize PFASs, with a heavy concentration in chemical manufacturing and resin production (Figure 5C).

## 4. Discussion

### 4.1. PFAS Regulatory Pattern in EU

Recent regulatory changes in the EU indicate a tightening of restrictions on PFASs, with a growing emphasis on a group-wide approach to regulation rather than targeting individual substances. In 2023, the European Chemicals Agency (ECHA) proposed a comprehensive restriction on the manufacture, use, and placement on the market of PFASs under REACH, aiming to significantly reduce emissions and environmental contamination [9]. Future trends point toward an increased focus on identifying safer alternatives to PFASs, enhancing monitoring and reporting requirements, and fostering international cooperation to address the global nature of PFAS pollution. These evolving regulatory landscapes underscore a commitment to mitigating the long-term environmental and health impacts of PFASs [9].

Beyond the overarching framework of the European Union’s REACH regulation, individual European countries have implemented their own regulatory measures to address the risks associated with PFASs. For instance, Denmark has introduced a ban on the use of PFASs in food contact materials, aiming to reduce potential exposure through food consumption [26]. The new order does not include a limitation on which substances are not in the scope of the PFAS chemical ban. It also does not specify which migration limits would be acceptable for the legitimacy of PFAS chemicals containing materials (https://www.sgs.com/en/news/2020/05/safeguards-07320-denmark-bans-pfas-chemicals-in-food-contact-paper-and-board, accessed on 18 November 2024). Similarly, the Netherlands has taken significant steps to monitor and regulate PFAS emissions, particularly from industrial sources, and has set stringent limits for PFAS concentrations in groundwater and drinking water. In 2023, Denmark, Germany, the Netherlands, Norway, and Sweden also formally submitted a restriction proposal for PFASs to the European Chemicals Agency, ECHA, under the chemical regulation REACH. The Committee for Risk Assessment (RAC) and the Committee for Socio-Economic Analysis (SEAC) have also been reviewing whether the proposed restrictions will meet the legal requirements of REACH since March 2023. The scientific evaluation of the proposal will begin once it is determined that the proposed restrictions meet legal requirements under REACH [9]. These national regulations complement EU-wide measures, reflecting the varying levels of concern and local environmental priorities across different member states.

### 4.2. PFAS Regulatory Pattern in North America

In the United States, the federal regulation of PFASs falls under the TSCA, which grants the USEPA authority to assess and manage chemical substances. Under the TSCA, the USEPA has taken significant steps to regulate PFASs, including issuing significant new use rules (SNURs) that require manufacturers and importers to notify the agency before resuming or beginning the use of certain long-chain PFASs [27]. The EPA is also proposing to designate PFOA and PFOS as hazardous substances under the Comprehensive Environmental Response, Compensation, and Liability Act (CERCLA), also commonly referred to as “Superfund”, which would require facilities to report releases that meet or exceed reportable quantities and allow the EPA to respond and require responsible parties to pay for the cost of remediation [28]. The agency has also prioritized the review of existing PFOA and PFOS chemicals, implemented drinking water health advisories, and proposed regulations to limit PFAS discharges into the environment [10]. Moreover, on October 2024, the USEPA issued the fifth Toxic Substances Control Act (TSCA) Test Order requiring testing on PFASs under the EPA’s National PFAS Testing Strategy [29]. These efforts are part of a broader strategy to reduce human and environmental exposure to PFASs, recognizing their persistence and potential health risks.

At the state level, California has emerged with some of the strictest consumer product regulations of PFASs in the US. For example, Assembly Bill 1200 (AB 1200) requires the labeling of chemicals, including PFASs, in cookware on a designated list created by the state as of 1 January 2024. A prohibition on the sale and distribution of plant fiber-based food packaging containing PFASs went into effect in January 2023. The law also prohibits advertising cookware as “PFAS-free” if the cookware contains PFASs [30]. Assembly Bill 1817 bans the manufacture, distribution, or sale of any new “textile articles” that contain “regulated PFAS” as of 1 January 2025 [31]. Proposition 65 also includes three PFAS chemicals for which companies are required to provide a “clear and reasonable warning” in a consumer product “known to the state to cause cancer or reproductive toxicity” [32]. Moreover, Assembly Bill 652 bars the manufacture, distribution, and sale of any new juvenile product containing PFASs, which was effective from 1 July 2023 [33]. Assembly Bill 2771 bans the manufacture, delivery, or sale of any cosmetic product containing “intentionally added” PFASs starting on 1 January 2025 [34]. For other states, Minnesota enacted the first prohibitions of products containing intentionally added PFASs under Amara’s Law starting in January 2025 [35]. Maine enacted several new sale prohibitions for products with intentionally added PFASs under their first act to stop PFAS pollution [36]. Vermont approved measures to regulate PFASs in certain goods in 2021, and the provisions in the new act have been implemented in phases, starting from July 2024 [37]. Maryland also passed the George “Walter” Taylor Act in 2021 to set restrictions on the use of products containing intentionally added PFAS chemicals. As of 1 January 2024, it is prohibited to use, manufacture, or knowingly sell or distribute Class B firefighting foam, certain rugs or carpets, and food packaging for direct food contact with intentionally added PFASs [38].

Canada has also implemented various regulatory measures to manage PFASs by recognizing their environmental persistence and potential human health risks. The CEPA serves as the primary framework for assessing and managing toxic substances including PFASs. While a number of PFASs (PFOS, PFOA, and long-chain (LC) PFCAs) are already regulated via the Prohibition of Certain Toxic Substances Regulations in 2012, the government published the final version of the Prohibition of Certain Toxic Substances in 2024 to further restrict the manufacture, use, sale, offer for sale, and import of the three PFAS subgroups that are already regulated [39]. Moreover, Environment and Climate Change Canada (ECCC) has conducted comprehensive assessments of PFASs, leading to targeted risk management strategies such as prohibiting certain PFASs in consumer products, environmental monitoring, and encouraging the development and use of safer alternatives [40]. These measures reflect Canada’s proactive approach to mitigate the risks associated with PFASs.

The North American Free Trade Agreement (NAFTA), now succeeded by the United States–Mexico–Canada Agreement (USMCA), has influenced PFAS regulation by facilitating the harmonization of environmental standards and regulatory cooperation among the three countries [41]. The NAFTA’s provisions for environmental cooperation have enabled collaborative efforts to address transboundary pollution issues, including PFAS contamination. Through mechanisms such as the Commission for Environmental Cooperation (CEC), the U.S., Canada, and Mexico have worked together on joint initiatives to monitor and manage PFAS pollution. This regional collaboration has enhanced the effectiveness of PFAS regulations by promoting information sharing, aligning regulatory approaches, and fostering joint research initiatives aimed at understanding and mitigating the impacts of PFASs across North America [41].

### 4.3. PFAS Regulatory Pattern in Asia–Pacific Region

In the Asia–Pacific region, countries like Japan, China, and South Korea have implemented various regulatory policies to manage PFASs, reflecting growing concerns over their environmental and health impacts. Japan has established regulations under its Chemical Substances Control Law (CSCL) to restrict the manufacture, use, and import of specific PFAS compounds, in which 138 PFAS compounds as Class I Specified Chemical Substances are prohibited for manufacture, import, and use, effective 10 January 2025. Designated substances include PFOA isomers and their salts and PFOA-related compounds including perfluorooctyl iodide (CAS 507-63-1) and 8:2 fluorotelomer alcohol (CAS 678-39-7) [42]. China, under its Ministry of Ecology and Environment, has included certain PFASs in its list of priority-controlled chemicals and mandated strict reporting and monitoring requirements. China’s Solid Waste and Chemicals Management Center (SCC) under the Ministry of Ecology and Environment (MEE) released the Reference List of PFOA, Its Salts and PFOA-related Compounds on 2 November 2023, in which 363 substances with CAS numbers and HS codes are included and subject to certain control measures [43]. South Korea has enacted regulations under its Act on Registration and Evaluation of Chemical Substances (K-REACH), focusing on the registration and evaluation of PFASs and imposing restrictions on their use. Under their Persistent Organic Pollutants (POPs) Control Act, there are restrictions/bans on PFOA, PFOS, and PFHxS. The POP measurement network includes three PFASs and an additional five PFASs (including PFBA, PFBS, PFHxA, PFNA, and PFDA). Also, some of the PFASs are designated and managed as “Toxic Substance” or “Substance subject to Intensive Control” under K-REACH [44]. These countries are progressively tightening controls on PFASs, aiming to mitigate their risks through comprehensive regulatory frameworks and industry compliance.

In addition, Asia–Pacific Economic Cooperation (APEC) plays a pivotal role in fostering regional collaboration in chemical safety and environmental protection, including the regulation of PFASs [45]. APEC provides a platform for its member economies to share best practices, harmonize standards, and coordinate efforts to address the challenges posed by PFASs. Through initiatives such as the Chemical Dialogue, APEC promotes the exchange of information on regulatory approaches, encourages the adoption of safer chemical management practices, and supports capacity-building activities to enhance regulatory frameworks across the region [45]. APEC’s collaborative efforts aim to ensure that member economies can effectively manage PFAS risks while facilitating trade and protecting public health and the environment.

Regulating PFASs in the Asia–Pacific region presents several challenges, including the diverse regulatory capacities and priorities among different countries, the need for robust scientific data on PFAS impacts, and the presence of extensive industrial uses of PFASs. Countries in the region vary significantly in their regulatory infrastructure and resources, leading to discrepancies in the enforcement and effectiveness of PFAS regulations. Additionally, the complexity and persistence of PFAS compounds require comprehensive scientific research to inform regulatory decisions, which can be resource-intensive. Industrial reliance on PFASs for various applications further complicates regulatory efforts, as transitioning to safer alternatives requires significant investment and technological advancements. Addressing these challenges necessitates enhanced regional cooperation, increased investment in scientific research, and the development of harmonized regulatory frameworks that balance economic and environmental considerations.

### 4.4. Environmental Impact and Current Regulations

PFASs (per- and polyfluoroalkyl substances) are a significant concern for water pollution due to their extreme persistence and mobility in aquatic environments. Our results also showed that in the Canada DSL database, compared to the categories of bioaccumulative (B; 18.8%) and ecotoxic (EcoT; 34.1%), as many as 81.3% of the listed substances are categorized as persistent (P) among all substances under CEPA categorization. Among all the regulatory databases assessed, only the Canada DSL had the environmental criteria and categorization listed under the authority, indicating the differences in focus on specific standards for different regulatory bodies. Although there was not a high percentage shown for substances with the bioaccumulative characteristic, PFASs are known for their ability to bioaccumulate in organisms and biomagnify through food webs, leading to elevated concentrations in top predators and posing significant ecological and health risks [46]. These chemicals can accumulate in the tissues of fish, birds, and mammals, where they persist due to their resistance to metabolic breakdown. As PFASs move up the food chain, their concentrations can increase, resulting in higher exposure levels for species at the top of the food web, including humans who consume contaminated fish and wildlife. This bioaccumulation and biomagnification can disrupt ecosystem balance and lead to adverse health effects in wildlife and humans, necessitating urgent regulatory and management interventions [46].

These chemicals, used in a variety of industrial and consumer products, can enter water bodies through industrial discharges, landfill leachates, and wastewater treatment plant effluents [47]. Once in water, PFASs can spread widely and resist degradation, leading to the contamination of rivers, lakes, and oceans. This persistent presence in water sources poses serious risks to aquatic life and human health, particularly when contaminated water is used for drinking [48]. PFAS contamination in soil and groundwater is a critical environmental issue, primarily resulting from the use of firefighting foams, industrial spills, and the application of contaminated biosolids as fertilizers. These chemicals can leach into the soil and migrate to groundwater, where they persist for extended periods due to their resistance to natural degradation processes [49]. Contaminated soil and groundwater can serve as long-term sources of PFASs, potentially affecting agricultural productivity and posing health risks through the consumption of contaminated crops and groundwater [50]. Addressing this contamination requires comprehensive strategies, including the identification and management of contamination sources, soil remediation technologies, and policies to prevent further contamination.

Effective environmental monitoring and management measures are crucial for addressing PFAS contamination. These measures include the systematic collection of data on PFAS concentrations in air, water, soil, and biota, as well as the identification of contamination sources. Advanced analytical techniques, such as high-resolution mass spectrometry, can be employed to detect and quantify PFASs at trace levels [51]. Management measures involve the implementation of best practices for industrial processes, the adoption of PFAS-free alternatives, and the development of risk-based guidelines for contaminated site management. Regular monitoring and reporting help track progress, ensure compliance with regulatory standards, and inform remediation efforts to mitigate the impact of PFASs on the environment and human health [12].

The remediation of PFAS-contaminated sites presents significant technological and logistical challenges due to the chemical’s persistence and resistance to conventional treatment methods [52]. Current remediation technologies include activated carbon adsorption, ion-exchange resins, advanced oxidation processes, and membrane filtration, each with varying degrees of effectiveness and feasibility [52,53,54,55]. Emerging techniques such as electrochemical oxidation, thermal treatment, and bioremediation are also being explored [52,56,57]. Despite these advancements, challenges remain in achieving complete PFAS removal, managing the disposal of PFAS-laden materials, and addressing the high costs and energy demands of treatment processes. Developing more efficient and sustainable remediation technologies, along with comprehensive site assessments and tailored remediation strategies, is essential to overcome these challenges and effectively mitigate PFAS contamination [58,59].

### 4.5. Industrial Impact and Challenges

Businesses face significant challenges in complying with PFAS regulations, including the complexity of monitoring and controlling emissions, the high costs of implementing new technologies, and the need to transition to safer alternatives. In addition, regulatory measures on PFASs have a substantial impact on international trade, as differing standards and restrictions can create barriers for businesses operating across borders. Countries with stringent PFAS regulations may restrict the import and export of PFAS-containing products, affecting global supply chains and market access. Companies must navigate complex regulatory landscapes, ensuring compliance with various national standards to maintain trade relationships. Harmonizing international regulations through agreements and collaborations can facilitate smoother trade, promote consistent environmental protection, and encourage the global adoption of safer chemicals and practices [60].

The U.S. Chamber of Commerce (USCC) performed an analysis by engaging with third-party experts to investigate the potential economic, fiscal, and environmental impacts of a ban on traded goods containing PFASs between the US and the EU. It was reported that the US and EU have the largest bilateral trade and investment relationship. The sectors with the highest value of imports from the EU to the US in 2022 were pharmaceutical (USD 111 billion) and automobile sectors (USD 37 billion) that rely heavily on PFAS applications. The proposed ECHA restriction would significantly disrupt global trade, and the need for the US to seek alternative trading partners would result in considerable business risks and increased transportation emissions because of greater shipping distances [61].

Practical challenges also stem from the absence of universally accepted testing methods and discrepancies in defining and classifying PFASs. The EU’s “essential use” concept attempts to limit PFAS use to applications in which their use is “necessary for health, safety, or is critical for the functioning of society” [62]. However, industries often face difficulties in identifying suitable alternatives due to the unique properties of PFASs. Furthermore, the lack of coordination among regulatory agencies can lead to inconsistent risk assessments and delayed responses to emerging data on PFAS toxicity and exposure [63,64]. Industries also face uncertainty in long-term compliance planning as regulatory landscapes evolve. Inconsistent implementation timelines and shifting standards can disrupt operations and increase costs. For example, companies operating across multiple jurisdictions must navigate divergent regulations, often resulting in a complex and resource-intensive compliance process. This challenge is exacerbated for small- and medium-sized enterprises (SMEs) that lack the capacity to monitor and adapt to regulatory changes effectively [65].

Furthermore, industries are frequently burdened by inadequate access to validated analytical methods and limited technical support. Many companies lack the in-house capability to conduct comprehensive PFAS testing, relying instead on third-party laboratories. Variability in testing methodologies can lead to discrepancies in compliance assessments and hinder regulatory enforcement. Establishing internationally recognized standards for PFAS detection and quantification would facilitate consistency and reliability in compliance evaluations [63]. The absence of clear guidance on phase-out strategies and alternative materials has posed challenges for industries seeking to transition away from PFASs. While some sectors have begun exploring non-fluorinated substitutes, concerns remain regarding the performance, safety, and cost-effectiveness of these alternatives. Collaborative research and public–private partnerships could accelerate the development of viable replacements, reducing the reliance on PFASs while maintaining product quality and safety [62].

### 4.6. Strategies and Possible Alternatives

At the current state, the tracing of PFASs is challenging since there is no legal requirement under REACH to provide the data of polymers or finished textile products through the supply chain. A lack of understanding and harmonization of PFAS definition and regulatory status among suppliers and manufacturers may also add more difficulties and hesitations among suppliers to disclose the proprietary formulations of their products. Regional disparities in the importance of PFAS transparency within the EU and globally have also hindered a unified effort to trace PFASs from suppliers to end users. To improve international harmonization, stakeholders could prioritize collaborative efforts in standardizing PFAS definitions, establishing uniform testing protocols, and promoting information exchange. Multilateral agreements, such as the Stockholm Convention on Persistent Organic Pollutants, could be expanded to address PFASs comprehensively. Additionally, capacity-building initiatives in low- and middle-income countries would strengthen regulatory enforcement. Industry partnerships can also facilitate the development of safer alternatives through joint research and innovation programs. Establishing an independent global PFAS monitoring network could further provide consistent data to inform decision-making and support regulatory alignment [62,65].

In addition, enhancing communication within the supply chain can be accomplished through voluntary initiatives such as updating supplier policies and building stronger relationships with suppliers. The incorporation of information on PFASs into ecolabeling and the use of technology, such as Radio-frequency identification (RFID) tags, could be adopted to provide greater transparency and visibility downstream. The enhancement of communication and transparency in information could avoid global ramifications possibly caused by regional restrictions of PFASs in the supply chain. Enhancing the engagement of industry in communications with authorities, academia, and the public can also lower the business risks associated with policy implementations. Businesses can also engage with regulatory agencies to stay informed about evolving requirements, participate in industry collaborations to share best practices, and invest in research and development to innovate and find effective PFAS substitutes. Collaborative efforts between regulatory bodies, industries, and academic institutions are critical to accelerating the development and adoption of PFAS alternatives. Establishing dedicated research funding, creating regulatory incentives, and facilitating knowledge-sharing platforms can drive progress in identifying safer substitutes. Additionally, fostering transparency in chemical safety data and conducting comprehensive lifecycle assessments will ensure the long-term sustainability of alternative materials [65].

Potential alternatives to PFASs are emerging, particularly in applications where their use is non-essential. Silicone-based coatings, plant-derived waxes, and biodegradable polymers are increasingly being explored as substitutes in textiles and food packaging. Additionally, advancements in nanotechnology and bio-based materials offer promise for the development of coatings and barriers with similar performance characteristics. However, further research is necessary to ensure that these alternatives meet durability, safety, and sustainability standards [63]. In the electronics and semiconductor industries, manufacturers are investigating fluorine-free etching and cleaning processes to reduce reliance on PFASs. Similarly, water-based and non-fluorinated firefighting foams have gained traction as replacements for aqueous film-forming foams (AFFFs) used in fire suppression. While these alternatives show potential, challenges remain in achieving equivalent fire-extinguishing performance in high-risk scenarios, requiring further innovation and testing [62].

### 4.7. Implications and Limitations

Despite growing recognition of the risks posed by PFASs, regulatory coverage and enforcement remain insufficient in many regions. Existing regulations often focus on a limited number of PFAS compounds, leaving many related chemicals unregulated. Furthermore, the enforcement of PFAS regulations can be inconsistent due to varying levels of regulatory capacity and resources among countries and regions. This insufficient coverage and enforcement hinder efforts to comprehensively manage PFAS risks and protect public health and the environment. Regulatory frameworks also face scientific and technical challenges, such as the development of reliable methods for detecting PFASs at low concentrations and an understanding of the cumulative effects of exposure to multiple PFASs. The lack of comprehensive toxicological data for many PFAS compounds further hinders the ability to establish safe exposure limits and enforce regulations effectively. Strengthening regulations, expanding their scope to cover more PFAS compounds, and ensuring consistent enforcement are essential steps toward more effective management of PFAS contamination.

The effective management of PFAS pollution requires international coordination and cooperation, given the global nature of PFAS production, use, and environmental impact. However, challenges arise from differing regulatory standards, varying levels of technical expertise, and divergent economic interests among countries. International cooperation is essential for harmonizing regulations, sharing scientific data, and developing global strategies to address PFAS contamination. Organizations such as the United Nations and the OECD can facilitate this coordination, but achieving consensus and implementing uniform standards remain challenging. Strengthening international collaboration through treaties, joint research initiatives, and shared best practices is crucial to overcoming these challenges and effectively managing PFASs on a global scale. While this study provides a comprehensive overview, it acknowledges limitations such as the variability in regulatory approaches and the rapidly changing nature of PFAS research. Future research should focus on developing advanced analytical techniques for detecting PFASs, understanding the mechanisms of PFAS toxicity, and exploring effective remediation strategies. Policymakers and industry stakeholders must stay informed about scientific advancements to make evidence-based decisions that protect public health and the environment.

## 5. Conclusions

The regulatory landscape for PFASs is diverse and evolving. Different countries and regions have adopted varying approaches to manage and mitigate the risks associated with PFASs. Among all the regulatory databases assessed, the Canada DSL is the only authority that has environmental criteria and categorizations for listed PFASs. The effective regulation of PFASs requires a multifaceted approach that includes stringent restrictions, continuous monitoring, and proactive risk assessment. In parallel with the evolving regulatory landscape, industry also faces multiple challenges including the absence of universally accepted testing methods, discrepancies in defining and classifying PFASs, and limited resources to develop alternatives. This study underscores the need for international cooperation and harmonized regulatory standards to address the transboundary nature of PFAS pollution. To improve international harmonization, stakeholders could prioritize standardizing PFAS definitions, establishing uniform testing protocols, and promoting information exchange. Expanding multilateral agreements like the Stockholm Convention and building capacity in low- and middle-income countries would strengthen regulatory enforcement. Industry partnerships and an independent global PFAS monitoring network could further support the development of safer alternatives and regulatory alignment. Collaborative efforts between regulatory bodies, industries, and academic institutions are essential for accelerating the development and adoption of PFAS alternatives. Establishing research funding, regulatory incentives, and knowledge-sharing platforms, along with fostering transparency in chemical safety data and conducting lifecycle assessments, will ensure the long-term sustainability of alternative materials. The global regulatory trends and impacts of PFASs highlight the complexity of managing these persistent chemicals. A coordinated effort involving regulatory agencies, industry, researchers, and the public is essential to mitigate the risks associated with PFASs and ensure a sustainable and healthy environment for future generations.

## Data Availability

Publicly available datasets were analyzed in this study. These data can be found here: https://australasia.setac.org/our-science-item/toward-a-new-comprehensive-global-database-of-per-and-polyfluoroalkyl-substances-pfass (accessed on 22 March 2025).
